# Quantitative and qualitative features of carotid and coronary atherosclerotic plaque among men and women

**DOI:** 10.3389/fcvm.2022.970438

**Published:** 2022-09-13

**Authors:** Carlotta Onnis, Christian Cadeddu Dessalvi, Filippo Cademartiri, Giuseppe Muscogiuri, Simone Angius, Francesca Contini, Jasjit S. Suri, Sandro Sironi, Rodrigo Salgado, Antonio Esposito, Luca Saba

**Affiliations:** ^1^Department of Radiology, Azienda Ospedaliero-Universitaria (A.O.U.), di Cagliari—Polo di Monserrato, Cagliari, Italy; ^2^Department of Medical Sciences and Public Health, Università Degli Studi di Cagliari, Cagliari, Italy; ^3^Department of Radiology, Fondazion Monasterio/CNR, Pisa, Italy; ^4^Department of Radiology, San Luca Hospital, Istituto Auxologico Italiano IRCCS, University of Milano-Bicocca, Milan, Italy; ^5^Stroke Diagnostic and Monitoring Division, AtheroPoint™, United States and Advanced Knowledge Engineering Centre, Global Biomedical Technologies Inc. (GBTI), Roseville, CA, United States; ^6^Department of Radiology, Antwerp University Hospital, Antwerp, Belgium; ^7^Experimental Imaging Center, Istituto di Ricovero e Cure a Carattere Scientifico (IRCCS) Ospedale San Raffaele, Milan, Italy; ^8^School of Medicine, Vita-Salute San Raffaele University, Milan, Italy

**Keywords:** atherosclerosis, sex-based differences, coronary imaging, carotid imaging, MRA (magnetic resonance angiography), CTA (computed tomographic angiography), gender

## Abstract

Cardiovascular diseases (CVDs), particularly ischemic heart disease (IHD) and stroke, present epidemiologically in a different way among sexes. The reasons of these sex-based differences should be delved into sex-specific cardiovascular (CV) risk factors and different mechanisms of atherosclerotic progression. Imaging techniques of both carotid and coronary atherosclerotic plaques represent a tool to demonstrate sex-related features which might be used to further and better assess CV risk of male and female population. The aim of this review is to evaluate current knowledge on sex-specific qualitative and quantitative plaque features of coronary and carotid atherosclerosis. We also discuss the clinical implication of a sex-based plaque phenotype, evaluated with non-invasive imaging techniques, such as CT-angiography and MRI-angiography, to stratify CV risk.

## Introduction: Patho-physiology of sex-related differences in atherosclerosis

Atherosclerosis is a progressive disease that affects arteries, characterized by the accumulation of lipids, macrophages, fibrous elements and smooth muscle cells within the vessel, forming the atherosclerotic plaque. Atherosclerosis is a chronic inflammatory condition that can lead to acute clinical events by plaque rupture and/or thrombosis, thus causing major events such as acute myocardial infarction (AMI), heart failure (HF), within the galaxy of ischemic heart disease (IHD), and stroke (1). These two cardiovascular diseases (CVDs) together represent the most common cause of death globally, with IHD and stroke responsible for 16 and 11% of the world’s total deaths, respectively, and that is why atherosclerosis is a condition of global interest (2, 3).

In the past decades, several studies have proven that atherosclerosis is strongly associated with several risk factors, such as hypertension, smoking, dyslipidemia, diabetes, obesity, age and family history, leading to the concept of vulnerable patient, at higher risk of developing acute CV events in the near future (4–6). Nonetheless, it is well known that CVDs presents differently among men and women: IHD in women develops after 7–10 years compared to men, in fact men are three times more likely to develop acute coronary syndromes (ACS) under the age of 60, while this tendency decreases with age and the likelihood among sexes equalizes over 75 years. With regards to cerebrovascular events, women over the age of 85 have a higher risk of developing stroke than men, leading to greater disability, mortality and case fatality in this group (7). Additionally, stroke is more likely to be the first manifestation of CVD in women, while IHD tends to be the first in men.

This knowledge has progressively led to the establishment of sex-specific CV risk assessment (8, 9), with sex referring to the biological characteristics of individuals as opposed to gender which indicates a broader concept rooted into society. Even though gender can influence health by reflecting the economic resources and healthcare access of the population, and despite the impossibility to define sex and gender in a binary way, the literature offers very few examples of non-binary trials or gender-related studies (10, 11). Thus, in this review we will use the term sex, but we emphasize its limitations.

Regarding sex-specific CV risk-factors, attention should be paid also to the modern role of women in the Western society: nowadays working patterns and activities are similar among sexes, but with women often having family responsibilities on top of time- and energy-consuming working roles. This joint social burden led to increased psychosocial stressors which further increase CV risk (12). Thus, modern female lifestyle and under-recognized risk factors, such as anxiety, depression, physical and psychological abuse, socioeconomic status and health literacy should be taken into consideration when adopting CV risk prevention strategies, especially during and after Sars-CoV-2 pandemic which exacerbated these risk factors (13).

The mechanisms underlying sex as a variable in atherosclerosis are constantly under-study, but the main finding is that until menopause women are protected by estrogens which seem to have an athero-protective role. In particular, estrogens have a pluripotent effect on the cardiovascular system, affecting the endothelium, coagulation, inflammation and adhesion (14). 17beta-estradiol (E2) is the most common form of circulating estrogen and by binding with estrogen receptors (ERs) it triggers a signaling cascade that alter gene expression affecting atherogenesis: For example, in hepatocytes the ER signaling activation is crucial to reverse cholesterol transport and protects against lipid accumulation in women. Furthermore, ER inhibits the proliferation of vascular smooth muscle cells in case of high level of glucose and, considering that those cells are a source of reactive oxygen species (ROS), which advances atherosclerosis, estrogen-ER complex performs its protective role. Finally, ER decreases differentiation of vascular muscle cells in osteoblastic-like cells, thus reducing occurrence of calcification within atherosclerotic lesions (15).

The important role of sex hormones also drove attention to sex hormone-binding globulin (SHBG), a protein that binds to and regulates available testosterone and estradiol. SHBG seems to be a potential risk stratification tool for predicting CV risk. In particular, there is an inverse association between serum SHBG levels and vascular risk factors (insulin resistance, diabetes, metabolic syndrome for example) and outcomes (IHD and stroke), which might be linked to either high free testosterone with consequent downstream pro-androgenic effects, or activation of inflammatory pathways (16, 17).

Moreover, proteomic studies have shown that female and male endothelial cell secretome responds differently to cellular stress induced by the same injury. Endothelial cells seem to adopt different strategies: in male more commonly apoptosis, while in female cells autophagy. Among the proteins secreted during apoptosis, PTX3 was found to have a crucial role in male-specific endothelial response to stressors. These results suggest a novel mechanism for sex-specific pathophysiological responses and identify PTX3 as a possible pharmacological target, considering its role as regulator of pro- and anti-inflammatory signals at the vascular bed, which should be further studied (18).

Female-specific risk factors, both modifiable and not, should also be taken into consideration. Adverse pregnancy outcomes (i.e., gestational hypertension, preterm delivery, preeclampsia/eclampsia) are associated with increased long-term CV risk of the mother and the mechanism seems to be an altered inflammatory state which affects maternal vasculature (19, 20). Lifetime estrogen exposure, early menarche and short reproductive life span in particular, represent another unmodifiable risk factor, as shown in a recent meta-analysis that highlights how a reproductive life span < 33 years is associated with higher rate of CVD events in midlife (21). Among the modifiable female-specific risk factors use of combined oral contraceptive and menopausal hormone therapy play an important role that is still under study (22). Even though systemic autoimmune disease is not a sex-specific risk factor, it should be taken into consideration that females are disproportionally affected, accounting for the 78% of patients (23). On one hand, chronic inflammation caused by autoimmune disease is associated with endothelial dysfunction and atherosclerosis progression, on the other hand steroid used to treat these conditions worsen hyperglycemia and dyslipidemia (24).

Clinical and pathological evaluation has revealed that males develop atherosclerotic plaque earlier, with the atherogenesis starting at a younger age in men than women; intima-media thickness (IMT) is usually greater in men until the age of 75, when we assist to the late catch-up phenomenon in women. Plaque inflammatory state seems to be greater in males than females and more vulnerable/unstable features are seen in non-invasive imaging of the male population. Moreover, plaque burden is greater in males while individual stenosis is greater in females, but plaque burden, associated with vulnerability, better predicts adverse ischemic events (25).

In this setting, non-invasive imaging techniques such as ultrasound, computed tomography angiography (CTA) and magnetic resonance angiography (MRA) can provide a useful tool to analyze plaque features among men and women. Thus, the aim of this review is to evaluate the current knowledge on sex-related differences of atherosclerosis in order to better stratify CV risk and target a preventive and effective therapy to the sub-population.

## State of the art: Quantitative and qualitative aspects of carotid and coronary atherosclerotic plaque among men and women

### Sex-related features in coronary atherosclerosis

**FIGURE 1:**
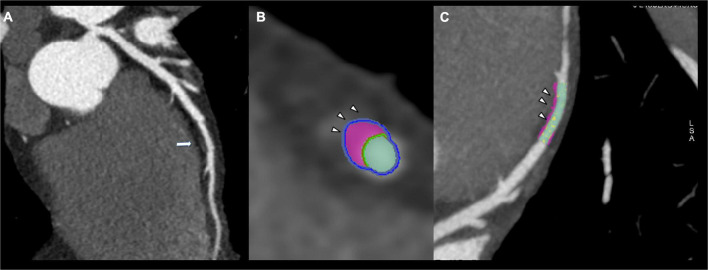
Thirty-six years old male showing fibrofatty plaque with positive remodeling on mid-lef anterior descending artery (arrow, **A**). Plaque analysis confirmed the fibrofatty composition of coronary plaque demonstrating the positive remodeling (arrowhead, **B,C**).

**FIGURE 2:**
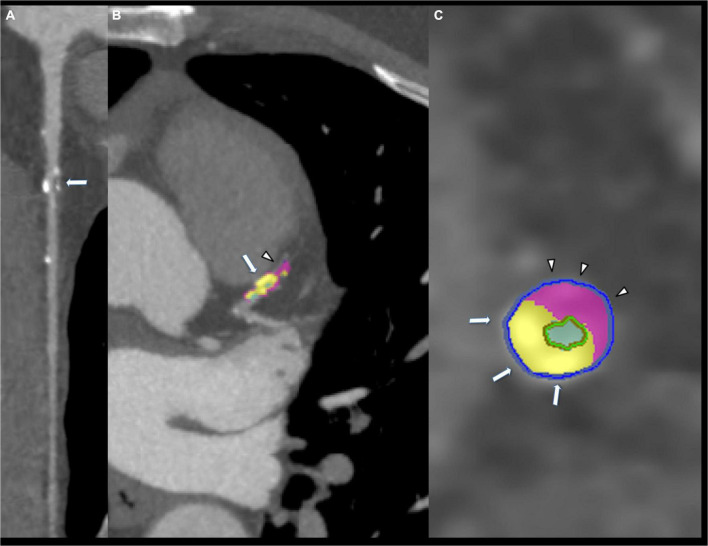
Eighty-five years old male showing mixed coronary plaque on proximal left anterior descending artery (arrow, **A**). Composition of plaque was composed by calcium (arrow, **B,C**) and fibrofatty portion (arrowhead **B,C**).

CTA has proven to be a valid technology that can easily identify patients at risk of subsequent CV events, and it can guide treatment management, improving outcome (Table 1). Recent studies have shown that a quantitative analysis of atherosclerotic plaque burden on cardiac-CTA (CCTA) can stratify the risk of future events better than traditional CV risk factors, coronary artery calcification (CAC) and coronary artery stenosis severity (26). In particular, low-attenuation plaque burden proved to be the strongest predictor of fatal or non-fatal myocardial infarction and with a low-attenuation plaque burden greater than 4% the risk of having a myocardial infarction is 5 times higher. Thus, CCTA adds important prognostic information but whether this assessment has an equal prognostic value in men and women is still under study. In fact, as Williams et al.(27) hypothesized based on the SCOT-HEART multicenter randomized controlled trial, sex differences in CAD may be explained by CT plaque assessment, which included evaluation of adverse plaque characteristics: positive remodeling, low-attenuation plaque, spotty calcification and the “napkin ring” sign (Figure 1). They discovered that women had lower CAC score, less frequent adverse plaque features, less obstructive CAD and overall lower quantitatively assessed plaque burden, leading to fewer subsequent MI compared to men. These findings, in agreement with the CONFIRM registry (28) and the ICONIC (29) study results, suggest that, while women presenting with stable chest pain have a specific, sex-based, plaque phenotype, symptomatic patients presenting with ACS have no sex difference in quantitative plaque features in culprit lesions. Indeed, there was no difference in necrotic core volume despite women having lower fibrous/fibrofatty plaque volume compared to men. Thus, even though women with stable angina presents with a different set of plaque features, when it comes to culprit lesions causing MI, both sexes seem to have similar plaque characteristics. Recent studies also showed that, within a given CAC score group, women tend to have smaller number, but larger size and density of calcified plaques compared to men, reflecting a more advanced atherosclerotic state/higher levels of inflammation, in line with less frequent obstructive disease but higher CVD mortality among women (mean age: 56.2 years) compared with men (30) (Figure 2).

**TABLE 1:** Studies regarding coronary atherosclerosis.

Authors	Number (patients)	Date published	Research objectives	Main results
Williams et al. (26) babi	1,769	2020	Role of non-calcified low-attenuation plaque burden assessed by coronary-CTA as a predictor of future risk of myocardial infarction (MI)	Low-attenuation plaque burden the strongest predictor of fatal or non-fatal MI (low-attenuation burden > 4% = nearly 5 times higher risk to have subsequent MI)
Williams et al. (27) babi	1,769	2021	Role of CCTA plaque assessment in explaining prognostic differences among men and women presenting with chest pain	Women (58 ± 9 years) less likely to have adverse plaque features compared to men and lower risk of subsequent MI
Schulman-Marcus et al. (28) babi	5,632	2016	Sex-specific associations between per-vessel CAD and major adverse CV events (MACE) over a 5-year period	Obstructive CAD more prevalent in men. Strong association between increased MACE risk and extent of per-vessel obstructive CAD
Conte et al. (29) babi	468	2021	Investigate sex and age differences in atherosclerotic features assessed by CCTA prior to acute coronary syndrome	Females had lower total plaque volume and fibrous/fibrofatty plaque volume within both the age groups
Shaw et al. (30) babi	63,215	2018	Sex differences in calcified plaque assessed by Agatson score and other CAC measures	Within CAC subgroups women had fewer calcified lesions and greater lesion size; CAC was associated with 1.3-fold higher risk for CV death among women; women with larger sized and more numerous CAC lesions had 2.2-fold higher CV mortality compared to men
El Mahdiui et al. (31) babi	211	2021	Role of sex and menopause on long-term plaque progression and evolution of plaque composition	Women had less fibrofatty atheroma volume on a per-lesion analysis; women < 55 years had more regression of fibrous and non-calcified atheroma volume over time compared to men
Lee et al. (33) babi	1,255	2020	Role of sex in total and compositional plaque volume progression in patients with CAD	9-year delay in women in developing total PV than in men; high-risk plaques more prevalent in men; women had greater calcified PV progression, slower non-calcified PV progression and less development of high-risk plaques
Xie et al. (36) babi	5,166	2017	Prognostic significance of non-obstructive left main CAD among sexes	Presence of non-obstructive LM plaque increased the risk for composite outcome and adverse events in women
Langabeer et al. (52) babi	16,861	2019	Gender differences in non-STEMI acute coronary syndrome	At baseline women were older and more often with history of prior CVD; women had higher in-hospital mortality, 23 min longer stay at ED, less likely to receive early invasive strategy compared to men
Langabeer et al. (53) babi	9,674	2018	Sex-related effects in outcomes in a large regional STEMI system of care	Length of stay was longer for women; females were less likely to survive at discharge and to be discharged to home

Additionally, El Mahdiui et al. (31) in their study evaluated plaque composition by serial CTA taking into consideration the influence of menopause. What they demonstrated is that men had more fibrofatty percentage atheroma volume (PAV), but pre-menopausal women, younger than 55 years, had greater regression of fibrous and non-calcified PAV over time compared to men of the same age, and given the positive association between non-calcified plaques with ischemia and ACS, these findings are in agreement with the lower risk of symptomatic CAD in young women (32). On the contrary, the lack of this regression in post-menopausal women suggests a progressive match to the non-calcified PAV of men with consequent increased risk of CAD in accordance with epidemiological data.

The PARADIGM study (33), a dynamic multinational observational registry of patients who underwent clinically indicated serial CCTA, also showed that total and compositional plaque volume (PV) progression rate differed among sexes: women had lower overall atherosclerotic burden in all age groups, reduced development of high-risk plaques and a PV progression mainly driven by calcified PV (faster calcified and slower non-calcified progression) while men had more non-calcified PV progression. The study demonstrated a 9-year delay in women for developing the total coronary atherosclerotic burden of men. The protective role of estrogen, until menopause, on development and progression have been suggested, especially taken into consideration the inhibitory effect of estrogen on vascular calcification (34). However, estrogen influence seems to be over-taken by the presence of risk factors resulting in worse outcome and prognosis for young women presenting with acute coronary syndrome compared to young men (35).

Finally, Xie et al. (36) found that non-obstructive left-main plaque was associated with 50% higher risk for adverse events among women, independently of CAD burden in other vessels, whereas this association was not significantly present among men; similarly, they found that women had a risk for future events 1.8-fold higher than men. Finding these sex-specific differences in prognosis provides an important risk and prognostic marker that should be considered during risk stratification of women. Thus, when evaluating prognosis, location of non-obstructive plaque should be considered.

New insights have been given by arterial and plaque characterization through intravascular ultrasound (IVUS), particularly concerning endothelial shear stress (ESS) which, with lower values, induces endothelial cell dysfunction and plaque progression. As Wentzel et al. (37) suggested in their study, which analyzed data from the PREDICTION study, coronary arteries and plaques were significantly smaller in females compared to males but ESS and ESS-related plaque progression were similar in both sexes. Only after stratifying for age they found that ESS-related plaque growth was more marked in women < 55 years and that female population showed a continuous reduction in magnitude of ESS-dependent plaque progression until 75 years of age, partially explaining the “catch up phenomenon” in female atherosclerosis.

### Sex-related features in carotid atherosclerosis

**FIGURE 3:**
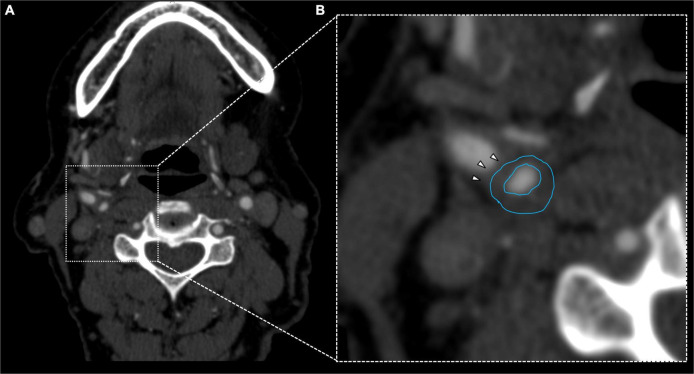
Fifty-six years old female showing fibrofatty plaque with concentric remodeling on right ICA **(A)**. Plaque analysis confirmed the fatty composition of the plaque (**B**, white arrowheads).

Epidemiology tells us that men have higher lifetime risk of stroke than women, but if women suffer from a stroke it is usually more severe, leading to greater disability (38). These epidemiological dissimilarities may have root in a sex-based pattern of carotid atherosclerosis, which is constantly under study especially through plaque imaging, including ultrasound and CTA, but mainly MRI (Table 2). It is well known that imaging biomarkers of vulnerability exist and that they predict stroke risk: intraplaque hemorrhage (IPH), best seen with MRI (39) but recently studied with CT as well (40); lipid-rich necrotic core (LRNC) either on CT and MRI; thin-rupture fibrous cap (TRFC), with contrast-enhanced MRI; carotid plaque thickness and surface morphology with all three imaging modalities; plaque volume with CT because of its high spatial resolution (41). These imaging features of vulnerability present differently among men and women. Indeed, men tend to have vulnerable plaques more frequently than women, as seen in the Rotterdam study (42) where they demonstrated that in a population with carotid wall thickening, IPH and LRNC, indicating plaque vulnerability, are highly frequent and more prevalent in men (Figure 3).

**TABLE 2:** Studies regarding carotid atherosclerosis.

Authors	Number (patients)	Date published	Research	Main results
Van den Bouwhuijsen et al. (42) babi	1,006	2012	Carotid plaque components as determinants of plaque progression and destabilization	Intraplaque hemorrhage (IPH) and lipid core (indicators of unstable plaque) more prevalent in men than women
Ota et al. (43) babi	131	2010	MRI carotid plaque assessment as a tool to demonstrate sex differences indicative of higher-risk plaque	Presence of thin/ruptured fibrous cap and lipid-rich necrotic core (LRNC) were more common in men; men had larger volumes of percent hemorrhage and necrotic core
Zhang et al. (44) babi	567	2021	To compare carotid atherosclerotic features among sexes	In both symptomatic and asymptomatic arteries, men had greater lumen, wall and total vessel area, higher mean wall thickness, higher prevalence of LRNC
Van Dam-Nolen et al. (45) babi	224	2022	To investigate sex differences in carotid plaque composition and morphology in patients with stroke	Total plaque volume was higher in men; IPH and LRNC more prevalent in men; men had more often coexistence of calcifications, LRNC and IPH, of thin/ruptured fibrous cap, LRNC and IPH and of all plaque features
Van Dam-Nolen et al. (46) babi	182	2021	To investigate the relation of lipoprotein(a) levels and carotid atherosclerotic plaque features	In women increased plasma Lp(a) was associated with IPH, in men with degree of stenosis
Schreiner et al. (47) babi	15,124	1996	To study the association of lipoprotein(a) with preclinical atherosclerotic disease in different race and gender groups	Lp(a) was associated with increased wall thickness in men while in women the association was stronger when smoking and diabetes were present
Song et al. (48) babi	189	2021	Sex differences in non-stenotic carotid plaque composition in patients with embolic stroke of undetermined source (ESUS)	Men had higher calcified plaque volume and IPH/LRNC ratio in carotid ipsilateral to stroke side; control cohort showed no sex difference in plaque volumes ipsilateral to stroke
Singh et al. (50) babi	906	2017	Age-specific sex differences in the presence of IPH	IPH was more prevalent in men for all ages; male sex modified the effect of age on the presence of IPH; with increasing age post-menopause, the odds of IPH in women become closer to that of men

Similarly, Ota et al. (43) assessed plaque characteristics in asymptomatic patients using 3-T MRI and they discovered that men present more frequently with LRNC, TRFC, larger percent volume of lipid core and IPH compared to women. With this features, atherosclerotic plaque is more prone to rupture, leading to subsequent ischemic events: hence, in their study Ota et al. suggested that 3-T carotid plaque MRI can identify high-risk phenotype and patients, and that their findings are consistent with epidemiological data, explaining why men age < 75 years have higher incidence of stroke than women.

Recently, in a study by Zhang et al. (44), both asymptomatic and symptomatic patients underwent 3-T carotid MRI. While in symptomatic carotid arteries, men showed similar plaque burden, despite larger vessel size, higher prevalence of LRNC and IPH compared to women, in asymptomatic carotid arteries men had more vulnerable features, such as higher percent LRNC and TRFC. Their findings are in agreement with the previous studies, indicating that asymptomatic male population tend to have vulnerable plaque features more frequently than women while plaque burden is similar in both sexes.

New insights have been given by the Plaque At Risk (PARISK) study (45), a prospective multicenter cohort study of patients with a high recurrent stroke risk with < 70% carotid stenosis. In particular, van Dam-Nolen et al. (46) examined patients with recent ischemic cerebrovascular symptoms and ipsilateral carotid stenosis < 70%, who underwent carotid MRI and CTA. They confirmed that men tend to have larger plaque volume, more vulnerable plaque composition (IPH, LRNC, TRFC) and more frequent coexistence of vulnerable features. They also suggested that sex-based plaque differences may not be related just to larger plaque burden in men: in fact, after adjusting the findings for total plaque volume, the prevalence of IPH and LRNC remained higher in men than in women, while the difference in IPH and LRNC volume disappeared, indicating that plaque burden does not fully explain the sex differences in carotid atherosclerosis and that sex plays an important role in the development of a vulnerable plaque rather than in the size of its components. This knowledge may lead to a sex-specific management of stroke and transient ischemic attack, with men benefitting from a carotid endarterectomy more than women (45).

Moreover, another work of van Dam-Nolen et al. (46) on the PARISK study, further evaluated the known importance of lipoprotein(a) as an independent risk factor for CVD and recurrent stroke (47). Not only they confirmed the positive association between Lp(a) levels and atherosclerosis, but also they suggested that Lp(a) distribution, as well as carotid plaque composition, differ between men and women and that Lp(a) levels peak during late perimenopause/postmenopause. Notably, they identified new associations between Lp(a) concentrations and plaque features: in male population elevated Lp(a) levels were associated with higher degree of stenosis, whilst in female population high Lp(a) levels tend to be associated with higher prevalence of IPH. This difference suggests that Lp(a) levels in women might be a stronger risk indicator for developing severe carotid atherosclerosis.

Additionally, Song et al. (48) examined sex differences in non-stenotic carotid plaque composition in patients with embolic stroke of undetermined source (ESUS) who underwent CTA. What they discovered is that in the atrial fibrillation cohort, used as control population, there was no significant sex difference in plaque volume and features, while among the ESUS cohort men had significantly higher IPH volume and IPH/LRNC ratio ipsilateral to stroke side compared to women, suggesting a differential contribution of atheroembolism from carotid plaque among men and women presenting with ESUS. Furthermore, they suggested IPH/LRNC ratio as a possible predictor of plaque rupture, stronger than LRNC alone, which may not always progress to rupture. In fact, IPH and increased neovascularity within a lipid-rich necrotic core can facilitate inflammation and core expansion, leading to greater rupture risk. Similarly, Saba et al. (49) observed that the ratio between IPH, indicated by Hounsfield units < 25 on CTA, and lipid volume is significantly associated with cerebrovascular events, hence representing a strong parameter for future events. Another study, by Singh et al. (50), also revealed that in patients with low-grade carotid stenosis the presence of IPH occurs more frequently with age, affecting less than 1% of patients before the age of 55 until a maximum of 12% by the age of 75 years, and with sex, affecting more men than women. An interesting result of this study was also the absence of IPH in females before the age of 65 years, which was progressively replaced with age, indicating that sex modifies the effect of age on carotid IPH. Hence, the infrequency of IPH in women before the age of 75 might explain the lower incidence of stroke among women of this age group when compared to men.

## Discussion

Atherosclerosis differs among sexes, both from a physio-pathological point of view and clinically as well. Epidemiology shows that women presents with IHD after an average of 7–10 years compared to men; men under the age of 60 are three times more likely to present with ACS, ST-segment elevation myocardial infarction (STEMI) and NSTEMI (51); while this tendency decreases over 75 years, we see an increase of morbidity and mortality rate in women, with 7.4% in-hospital mortality for STEMI in women vs. 4.6% in men and 4.8 vs. 3.9%, respectively, for NSTEMI (52, 53). Moreover, women over the age of 85 have a higher risk of developing stroke than men, with 55,000 more females having a stroke than males every year, indicating that women might be treated less aggressively in primary and secondary prevention for this disease (7). Other data from the Heart Disease and Stroke Statistics—2022 Update (54) revealed that stroke is more prevalent in men until the age of 80 years, but over 80 this tendency inverts, and that more females than men die of stroke each year, indicating that the cause might be this higher prevalence in elderly women.

What the literature reveals is that these epidemiological disparities have a physio-pathological counterpart which can be studied histologically and with invasive methods, but also with non-invasive techniques. In this context, radiology with CTA, MRA and ultrasound provides useful tools that may be used not only for academic purposes but also for clinical ones, giving information regarding plaque characteristics that can be linked to outcome and prognosis (Table 3). Indeed, CTA provides information regarding plaque features of vulnerability (positive remodeling, low-attenuation plaque, spotty calcification, “napkin ring” sign) and thus prognosis. In fact, low-attenuation plaque volume (26) and IPH/LRNC ratio (49) appeared to be two strong prognostic markers that can predict fatal and non-fatal future MI the former, and cerebrovascular events the latter. In addition, through CCTA it is possible to understand the female-specific coronary plaque phenotype, which partially explains the epidemiological data mentioned above. Women before menopause tend to have lower CAC score, less high-risk plaque features, less plaque volume, less obstructive CAD (27) and greater fibrous and non-calcified PAV regression (31). After menopause, the literature seems to identify a different female-specific coronary plaque phenotype, which tends to be more similar to that one of men: Less non-calcified PAV regression (31) and smaller number but larger size and higher density of calcified plaque (30), progressively matching, with a 9-year delay, the total coronary atherosclerotic burden of men (33). Additionally, taken into consideration that non-calcified PV seems to be influenced mainly by environmental factors, while CAC and calcified PV are linked to genetics (55), CCTA might be a useful tool to early detect non-calcified plaque in order to start an early lifestyle intervention, thus preventing coronary plaque formation.

**TABLE 3:** Difference between men and women of high-risk plaque features in carotid and coronary arteries.

High-risk features	Prevalence	References
**Carotid arteries**		
IPH	Men > Women	(42)
LRNC	Men > Women	(42–43)
Thin/ruptured fibrous cap	Men > Women	(43)
Wall thickness	Men > Women	(44)
Total vessel area	Men > Women	(45)
Total plaque volume	Men > Women	(46)
Calcified plaque volume	Men > Women	(48)
**Coronary arteries**		
Low-attenuation burden > 4%	Men > Women	(26–27)
Extent of per-vessel obstructive CAD	Men > Women	(28)
Total plaque volume	Men > Women	(29)
Fibrous/fibrofatty plaque volume	Men > Women	(29–31)
Larger sized and number of calcified lesions	Men < Women	(30)

Notably, the literature (27) shows that women presents with this specific, sex-based plaque phenotype more evidently when they are asymptomatic or in case of stable angina. Indeed, the above-mentioned sex-based features tend to flatten when ACS occurs, and no significant difference among men and women can be found in culprit lesions causing MI. The necrotic core volume is in fact similar among sexes. This finding might suggest the role of CCTA for risk stratification, before ACS occur, and the importance of primary prevention in order to treat aggressively those women presenting with high-risk features or markers of worse prognosis. With this regard, an interesting insight has been given by Xie et al. (36) who suggested that location of CAD should be assessed with CCTA in order to stratify the risk of future events in women: particularly, they found that non-obstructive left main plaque has a great prognostic implication, with a 50% higher risk for females of having adverse events, independently from CAD burden in other vessels.

Finally, a female-specific plaque phenotype can be seen in CT/MRI evaluation of carotid atherosclerosis as well. Women tend to have less features of vulnerability compared to men: less IPH, LRNC and calcified PV in particular, in accordance with a less frequent tendency to rupture compared to male carotid plaques (42, 43). Thus, CT and MRI represent valid techniques that can detect high-risk features, in order to discriminate which group of patients might benefit from an aggressive primary prevention therapy. Moreover, the ratio of IPH/LRNC seems to be a predictive marker of future plaque rupture and consequent cerebrovascular events, and various studies showed that the ratio is usually higher in the male population (48, 49). Ultrasound is worth mentioning because of its availability and cost feasibility; in addition, it offers accurate and reproducible imaging evaluation of lumen and vessel wall, particularly IMT. However, it has a low sensitivity and specificity for detection of other plaque features, such as fibrous cap, ulceration, plaque inflammation, IPH and LRNC (56). Ultrasound does not distinguish IPH and LRNC because they both appear hypoechogenic, making it difficult to evaluate the ratio, and it is affected by calcified components that create acoustic shadow. For these reasons, ultrasound is not useful for the assessment of most quantitative measurements of carotid plaque and it is not considered the imaging modality of choice (41).

As suggested by Nasir et al. (57) the question is how and if this information will modify medical management, especially if the assessment of plaque phenotype will lead to consideration of a lipid-lowering therapy of various intensities and/or whether the presence of high-risk plaque characteristics will influence the decision-making process of starting antiplatelet treatment.

Further studies should be carried forward in order to fully comprehend how these insights can impact the clinical management of atherosclerosis in men and women, but what we know so far from the literature is that there are undeniable differences among sexes in atherosclerotic presentation, that these phenotypes can be successfully evaluated throughout non-invasive imaging techniques and that this type of plaque assessment should be strengthened and enhanced because of its prognostic implication.

## Author contributions

LS, CCD, and AE contributed to the conception and design of the study. CO and FCa wrote the first draft of the manuscript. GM, SA, JS, FC, SS, and RS wrote sections of the manuscript. All authors contributed to the manuscript revision, read, and approved the submitted version.

## References

[1] Lusis AJ. Atherosclerosis. *Nature.* (2000) 407:233–41. 10.1038/35025203 11001066 PMC2826222

[2] GBD 2019 Diseases and Injuries Collaborators. Global burden of 369 diseases and injuries in 204 countries and territories, 1990-2019: A systematic analysis for the global burden of disease study 2019. *Lancet.* (2020) 396:1204–22. 10.1016/S0140-6736(20)30925-9 33069326 PMC7567026

[3] World Health Organization. *The top 10 causes of death.* Geneva: World Health Organization (2020).

[4] Naghavi M Libby P Falk E Casscells SW Litovsky S Rumberger J From vulnerable plaque to vulnerable patient: A call for new definitions and risk assessment strategies: Part I. *Circulation.* (2003) 108:1664–72. 10.1161/01.CIR.0000087480.94275.97 14530185

[5] Naghavi M Libby P Falk E Casscells SW Litovsky S Rumberger J From vulnerable plaque to vulnerable patient: A call for new definitions and risk assessment strategies: Part II. *Circulation.* (2003) 108:1772–8. 10.1161/01.CIR.0000087481.55887.C9 14557340

[6] Yusuf S Hawken S Ounpuu S Dans T Avezum A Lanas F Effect of potentially modifiable risk factors associated with myocardial infarction in 52 countries (the INTERHEART study): Case-control study. *Lancet.* (2004) 364:937–52. 10.1016/S0140-6736(04)17018-9 15364185

[7] Persky RW Turtzo LC McCullough LD. Stroke in women: Disparities and outcomes. *Curr Cardiol Rep.* (2010) 12:6–13. 10.1007/s11886-009-0080-2 20425178 PMC2861793

[8] SCORE2 Working Group and ESC Cardiovascular Risk Collaboration. SCORE2 risk prediction algorithms: New models to estimate 10-year risk of cardiovascular disease in Europe. (2021) 42:2439–54. 10.1093/eurheartj/ehab309 34120177 PMC8248998

[9] Maffei S Guiducci L Cugusi L Cadeddu C Deidda M Gallina S Women-specific predictors of cardiovascular disease risk – new paradigms. *Int J Cardiol.* (2019) 286:190–7. 10.1016/j.ijcard.2019.02.005 30803890

[10] Pelletier R Khan NA Cox J Daskalopoulou SS Eisenberg MJ Bacon SL Sex versus gender-related characteristics: Which predicts outcome after acute coronary syndrome in the young? *J Am Coll Cardiol.* (2016) 67:127–35. 10.1016/j.jacc.2015.10.067 26791057

[11] Norris CM Johnson NL Hardwicke-Brown E McEwan M Pelletier R Pilote L. The contribution of gender to apparent sex differences in health status among patients with coronary artery disease. *J Womens Health (Larchmt).* (2017) 26:50–7. 10.1089/jwh.2016.5744 27400270

[12] Sciomer S Moscucci F Maffei S Gallina S Mattioli AV. Prevention of cardiovascular risk factors in women: The lifestyle paradox and stereotypes we need to defeat. *Eur J Prev Cardiol.* (2019) 26:609–10. 10.1177/2047487318810560 30373379

[13] Vogel B Acevedo M Appelman Y Bairey Merz CN Chieffo A Figtree GA The lancet women and cardiovascular disease commission: Reducing the global burden by 2030. *Lancet.* (2021) 397:2385–438. 10.1016/S0140-6736(21)00684-X 34010613

[14] Baker L Meldrum KK Wang M Sankula R Vanam R Raiesdana A The role of estrogen in cardiovascular disease. *J Surg Res.* (2003) 115:325–44. 10.1016/s0022-4804(03)00215-4 14697301

[15] Aryan L Younessi D Zargari M Banerjee S Agopian J Rahman S The role of estrogen receptors in cardiovascular disease. *Int J Mol Sci.* (2020) 21:4314. 10.3390/ijms21124314 32560398 PMC7352426

[16] Madsen TE Luo X Huang M Park KE Stefanick ML Manson JE Circulating SHBG (sex hormone-binding globulin) and risk of ischemic stroke: Findings from the WHI. *Stroke.* (2020) 51:1257–64. 10.1161/STROKEAHA.120.028905 32078494 PMC7144884

[17] Rexrode KM Manson JE Lee IM Ridker PM Sluss PM Cook NR Sex hormone levels and risk of cardiovascular events in postmenopausal women. *Circulation.* (2003) 108:1688–93. 10.1161/01.CIR.0000091114.36254.F3 12975257

[18] Cattaneo MG Banfi C Brioschi M Lattuada D Vicentini LM. Sex-dependent differences in the secretome of human endothelial cells. *Biol Sex Differ.* (2021) 12:7. 10.1186/s13293-020-00350-3 33413676 PMC7791663

[19] Søndergaard MM Hlatky MA Stefanick ML Vittinghoff E Nah G Allison M Association of adverse pregnancy outcomes with risk of atherosclerotic cardiovascular disease in postmenopausal women. *JAMA Cardiol.* (2020) 5:1390–8. 10.1001/jamacardio.2020.4097 32936228 PMC7495331

[20] O’Kelly AC Michos ED Shufelt CL Vermunt JV Minissian MB Quesada O Pregnancy and reproductive risk factors for cardiovascular disease in women. *Circ Res.* (2022) 130:652–72. 10.1161/CIRCRESAHA.121.319895 35175837 PMC8870397

[21] Rexrode KM Madsen TE Yu AYX Carcel C Lichtman JH Miller EC. The impact of sex and gender on stroke. *Circ Res.* (2022) 130:512–28. 10.1161/CIRCRESAHA.121.319915 35175851 PMC8890686

[22] Mishra SR Chung HF Waller M Dobson AJ Greenwood DC Cade JE Association between reproductive life span and incident nonfatal cardiovascular disease: A pooled analysis of individual patient data from 12 studies. *JAMA Cardiol.* (2020) 5:1410–8. 10.1001/jamacardio.2020.4105 32936210 PMC7495334

[23] Fairweather D Frisancho-Kiss S Rose NR. Sex differences in autoimmune disease from a pathological perspective. *Am J Pathol.* (2008) 173:600–9. 10.2353/ajpath.2008.071008 18688037 PMC2527069

[24] Young L Cho L. Unique cardiovascular risk factors in women. *Heart.* (2019) 105:1656–60. 10.1136/heartjnl-2018-314268 31315936

[25] Man JJ Beckman JA Jaffe IZ. Sex as a biological variable in atherosclerosis. *Circ Res.* (2020) 126:1297–319. 10.1161/CIRCRESAHA.120.315930 32324497 PMC7185045

[26] Williams MC Kwiecinski J Doris M McElhinney P D’Souza MS Cadet S Low-attenuation noncalcified plaque on coronary computed tomography angiography predicts myocardial infarction: Results from the multicenter SCOT-HEART trial (Scottish computed tomography of the heart). *Circulation.* (2020) 141:1452–62. 10.1161/CIRCULATIONAHA.119.044720 32174130 PMC7195857

[27] Williams MC Kwiecinski J Doris M McElhinney P D’Souza MS Cadet S Sex-Specific computed tomography coronary plaque characterization and risk of myocardial infarction. *JACC Cardiovasc Imaging.* (2021) 14:1804–14. 10.1016/j.jcmg.2021.03.004 33865779 PMC8435010

[28] Schulman-Marcus J Hartaigh BÓ Gransar H Lin F Valenti V Cho I Sex-specific associations between coronary artery plaque extent and risk of major adverse cardiovascular events: The CONFIRM long-term registry. *JACC Cardiovasc Imaging.* (2016) 9:364–72. 10.1016/j.jcmg.2016.02.010 27056154 PMC5039939

[29] Conte E Dwivedi A Mushtaq S Pontone G Lin FY Hollenberg EJ Age- and sex-related features of atherosclerosis from coronary computed tomography angiography in patients prior to acute coronary syndrome: Results from the ICONIC study. *Eur Heart J Cardiovasc Imaging.* (2021) 22:24–33. 10.1093/ehjci/jeaa210 32793985 PMC8218779

[30] Shaw LJ Min JK Nasir K Xie JX Berman DS Miedema MD Sex differences in calcified plaque and long-term cardiovascular mortality: Observations from the CAC Consortium. *Eur Heart J.* (2018) 39:3727–35. 10.1093/eurheartj/ehy534 30212857 PMC6209852

[31] El Mahdiui M Smit JM van Rosendael AR Neglia D Knuuti J Saraste A Sex differences in coronary plaque changes assessed by serial computed tomography angiography. *Int J Cardiovasc Imaging.* (2021) 37:2311–21. 10.1007/s10554-021-02204-4 33694122 PMC8286938

[32] Chang HJ Lin FY Lee SE Andreini D Bax J Cademartiri F Coronary atherosclerotic precursors of acute coronary syndromes. *J Am Coll Cardiol.* (2018) 71:2511–22. 10.1016/j.jacc.2018.02.079 29852975 PMC6020028

[33] Lee SE Sung JM Andreini D Al-Mallah MH Budoff MJ Cademartiri F Sex differences in compositional plaque volume progression in patients with coronary artery disease. *JACC Cardiovasc Imaging.* (2020) 13:2386–96. 10.1016/j.jcmg.2020.06.034 32828763

[34] Osako MK Nakagami H Koibuchi N Shimizu H Nakagami F Koriyama H Estrogen inhibits vascular calcification via vascular RANKL system: Common mechanism of osteoporosis and vascular calcification. *Circ Res.* (2010) 107:466–75. 10.1161/CIRCRESAHA.110.216846 20595654

[35] Chandrasekhar J Mehran R. Sex-based differences in acute coronary syndromes: Insights from invasive and noninvasive coronary technologies. *JACC Cardiovasc Imaging.* (2016) 9:451–64. 10.1016/j.jcmg.2016.02.004 27056164

[36] Xie JX Eshtehardi P Varghese T Goyal A Mehta PK Kang W Prognostic significance of nonobstructive left main coronary artery disease in women versus men: Long-term outcomes from the CONFIRM (coronary CT angiography evaluation for clinical outcomes: An international multicenter) registry. *Circ Cardiovasc Imaging.* (2017) 10:e006246. 10.1161/CIRCIMAGING.117.006246 28790123 PMC5663295

[37] Wentzel JJ Papafaklis MI Antoniadis AP Takahashi S Cefalo NV Cormier M Sex-related differences in plaque characteristics and endothelial shear stress related plaque-progression in human coronary arteries. *Atherosclerosis.* (2022) 342:9–18. 10.1016/j.atherosclerosis.2021.12.013 34999306

[38] Appelros P Stegmayr B Terént A. Sex differences in stroke epidemiology: A systematic review. *Stroke.* (2009) 40:1082–90. 10.1161/STROKEAHA.108.540781 19211488

[39] Schindler A Schinner R Altaf N Hosseini AA Simpson RJ Esposito-Bauer L Prediction of stroke risk by detection of hemorrhage in carotid plaques: Meta-analysis of individual patient data. *JACC Cardiovasc Imaging.* (2020) 13:395–406. 10.1016/j.jcmg.2019.03.028 31202755

[40] Saba L Francone M Bassareo PP Lai L Sanfilippo R Montisci R CT attenuation analysis of carotid intraplaque hemorrhage. *AJNR Am J Neuroradiol.* (2018) 39:131–7. 10.3174/ajnr.A5461 29191874 PMC7410713

[41] Saba L Saam T Jäger HR Yuan C Hatsukami TS Saloner D Imaging biomarkers of vulnerable carotid plaques for stroke risk prediction and their potential clinical implications. *Lancet Neurol.* (2019) 18:559–72. 10.1016/S1474-4422(19)30035-3 30954372

[42] van den Bouwhuijsen QJ Vernooij MW Hofman A Krestin GP van der Lugt A Witteman JC. Determinants of magnetic resonance imaging detected carotid plaque components: The Rotterdam Study. *Eur Heart J.* (2012) 33:221–9. 10.1093/eurheartj/ehr227 21821844

[43] Ota H Reeves MJ Zhu DC Majid A Collar A Yuan C Sex differences in patients with asymptomatic carotid atherosclerotic plaque: In vivo 3.0-T magnetic resonance study. *Stroke.* (2010) 41:1630–5. 10.1161/STROKEAHA.110.581306 20616325

[44] Zhang L Zhu L Lu M Zhao X Li F Cai J Comparison of carotid plaque characteristics between men and women using magnetic resonance vessel wall imaging: A Chinese atherosclerosis risk evaluation study. *J Magn Reson Imaging.* (2021) 54:646–54. 10.1002/jmri.27576 33638575

[45] van Dam-Nolen DHK van Egmond NCM Dilba K Nies K van der Kolk AG Liem MI Sex differences in plaque composition and morphology among symptomatic patients with mild-to-moderate carotid artery stenosis. *Stroke.* (2022) 53:370–8. 10.1161/STROKEAHA.121.036564 34983237 PMC8785521

[46] van Dam-Nolen DHK van Dijk AC Crombag GAJC Lucci C Kooi ME Hendrikse J Lipoprotein(a) levels and atherosclerotic plaque characteristics in the carotid artery: The plaque at RISK (PARISK) study. *Atherosclerosis.* (2021) 329:22–9. 10.1016/j.atherosclerosis.2021.06.004 34216874

[47] Schreiner PJ Heiss G Tyroler HA Morrisett JD Davis CE Smith R. Race and gender differences in the association of Lp(a) with carotid artery wall thickness. The atherosclerosis risk in communities (ARIC) study. *Arterioscler Thromb Vasc Biol.* (1996) 16:471–8. 10.1161/01.atv.16.3.471 8630675

[48] Song JW Cao Q Siegler JE Thon JM Woo JH Cucchiara BL. Sex differences in carotid plaque composition in patients with embolic stroke of undetermined source. *J Am Heart Assoc.* (2021) 10:e020143. 10.1161/JAHA.120.020143 33904317 PMC8200747

[49] Saba L Micheletti G Brinjikji W Garofalo P Montisci R Balestrieri A Carotid intraplaque-hemorrhage volume and its association with cerebrovascular events. *AJNR Am J Neuroradiol.* (2019) 40:1731–7. 10.3174/ajnr.A6189 31558503 PMC7028561

[50] Singh N Moody AR Zhang B Kaminski I Kapur K Chiu S Age-specific sex differences in magnetic resonance imaging-depicted carotid intraplaque hemorrhage. *Stroke.* (2017) 48:2129–35. 10.1161/STROKEAHA.117.017877 28706117 PMC5606979

[51] Vakhtangadze T Singh Tak R Singh U Baig MS Bezsonov E. Gender differences in atherosclerotic vascular disease: From lipids to clinical outcomes. *Front Cardiovasc Med.* (2021) 8:707889. 10.3389/fcvm.2021.707889 34262956 PMC8273377

[52] Langabeer JR II Champagne-Langabeer T Fowler R Henry T. Gender-based outcome differences for emergency department presentation of non-STEMI acute coronary syndrome. *Am J Emerg Med.* (2019) 37:179–82. 10.1016/j.ajem.2018.05.005 29754965

[53] Langabeer JR II Henry TD Fowler R Champagne-Langabeer T Kim J Jacobs AK. Sex-based differences in discharge disposition and outcomes for ST-segment elevation myocardial infarction patients within a regional network. *J Womens Health (Larchmt).* (2018) 27:1001–6. 10.1089/jwh.2017.6553 29319393

[54] Tsao CW Aday AW Almarzooq ZI Alonso A Beaton AZ Bittencourt MS Heart disease and stroke statistics-2022 update: A report from the American heart association. *Circulation.* (2022) 145:e153–639. 10.1161/CIR.0000000000001052 35078371

[55] Drobni ZD Kolossvary M Karady J Jermendy AL Tarnoki AD Tarnoki DL Heritability of coronary artery disease: Insights from a classical twin study. *Circ Cardiovasc Imaging.* (2022) 15:e013348. 10.1161/CIRCIMAGING.121.013348 35290075 PMC8925867

[56] Spanos K Tzorbatzoglou I Lazari P Maras D Giannoukas AD. Carotid artery plaque echomorphology and its association with histopathologic characteristics. *J Vasc Surg.* (2018) 68:1772–80. 10.1016/j.jvs.2018.01.068 29803682

[57] Nasir K Sharma G Blumenthal RS. Sex differences in coronary plaque composition and progression: Will it influence clinical management? *JACC Cardiovasc Imaging.* (2020) 13:2397–9. 10.1016/j.jcmg.2020.05.040 32828764

